# Is Network Clustering Detectable in Transmission Trees?

**DOI:** 10.3390/v3060659

**Published:** 2011-06-03

**Authors:** David Welch

**Affiliations:** Department of Statistics, Pennsylvania State University, 325 Thomas Building, University Park, PA 16802, USA; E-Mail: jdw21@stat.psu.edu; Tel.: +1-814-865-1348

**Keywords:** network, contact network, clustering, simulations, SEIR, epidemics, transmission tree, population structure

## Abstract

Networks are often used to model the contact processes that allow pathogens to spread between hosts but it remains unclear which models best describe these networks. One question is whether clustering in networks, roughly defined as the propensity for triangles to form, affects the dynamics of disease spread. We perform a simulation study to see if there is a signal in epidemic transmission trees of clustering. We simulate susceptible-exposed-infectious-removed (SEIR) epidemics (with no re-infection) over networks with fixed degree sequences but different levels of clustering and compare trees from networks with the same degree sequence and different clustering levels. We find that the variation of such trees simulated on networks with different levels of clustering is barely greater than those simulated on networks with the same level of clustering, suggesting that clustering can not be detected in transmission data when re-infection does not occur.

## 1. Introduction

To understand the dynamics of infectious diseases it is crucial to understand the structure and interactions within the host population. Conversely, it is possible to learn something about host population structure by observing the pattern of pathogen spread within it. In either case, it is necessary to have a good model of the host population structure and interactions within it. Networks, where nodes of the network represent hosts and edges between nodes represent contacts across which pathogens may be transmitted, are now regularly used to model host interactions [1–3]. While many models have been proposed to describe the structure of these contact networks for different populations and different modes of transmission, it is not yet understood how different features of networks affect the spread of pathogens.

One promising development in this field is the use of statistical techniques which aim to model a contact network based on data relating to the passage of a pathogen through a population. Such data includes infection times [4–6] and genetic sequences that are collected from an epidemic present in the population of interest [7–9]. These data have previously been shown to be useful for reconstructing transmission histories (the distinction between a contact network and a transmission history is that a contact network includes all edges between hosts across which disease may spread, whereas the transmission history is just the subset of edges across which transmission actually occurred). Infection times can be used to crudely reconstruct transmission histories by examining which individuals were infectious at the time that any particular individual was infected [10]. Genetic sequences from viruses are informative about who infected whom by comparing the similarity between sequences. Due to the random accumulation of mutations in the sequences, we expect sequences from an infector/infectee pair to be much closer to each other than sequences from a randomly selected pair in the population (see [11] for a review of modern approaches to analysing viral genetic data). The work of [4–6] seeks to extend the use of this data to reconstruct a model for the whole contact network rather than just the transmission history. In theory, these statistical methods could settle arguments about which features of the network are important in the transmission of the disease and which are simply artifacts of the physical system.

**Figure 1.**
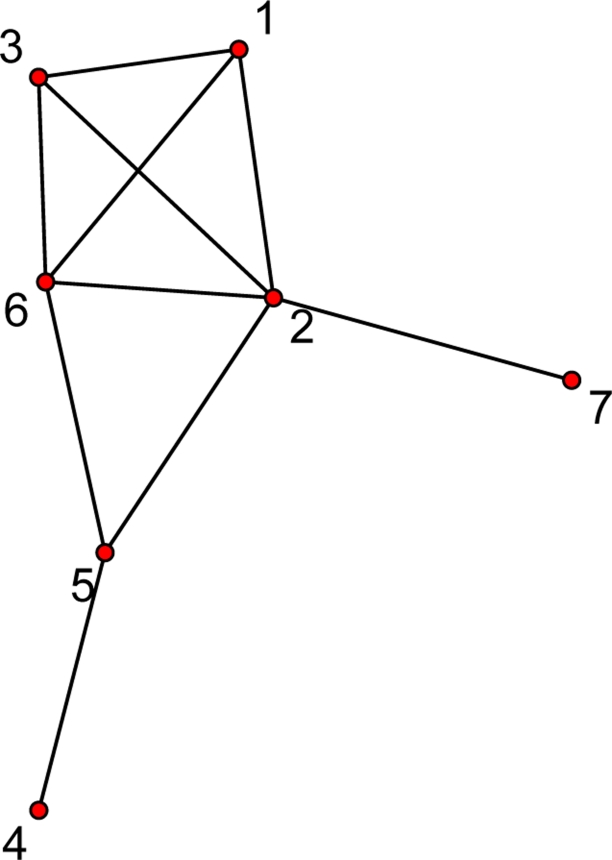
An example of a network on 7 nodes. The *nodes* are the red dots, labelled 1 to 7 and represent individuals in the population. The *edges* are shown as black lines connecting the nodes and represent possible routes of transmission. The *degree* of each node is number of edges adjacent to it, so that node 5 has degree 3 and node 7 has degree 1. The *degree sequence* of the network is the count of nodes with a given degree and can be represented by the vector (0*,* 2*,* 0*,* 3*,* 1*,* 1) showing that there are 0 nodes of degree 0, 2 of degree 1, 0 of degree 2 and so on. A *cycle* in the network is a path starting at a node and following distinct edges to end up back at the same node. For example, the path from node 6 to node 1 to node 3 and back to node 6 is a cycle but there is no cycle that includes node 4. *Clustering* is a measure of propensity of cycles of length 3 (*triangles*) to form. Here, the edges (2,1) and (2,6) form a triangle with the edge (1,6), so work to increase clustering in the network. However, the edges (2,1) and (2,5) do not comprise part of a triangle as (1,5) does not exist, so work to decrease clustering.

In this article, we focus on clustering in networks and ask whether or not networks which differ only in their level of clustering could be distinguished if all we observed was transmission data from an epidemic outbreak. The answer to this question will determine whether these new statistical techniques can be extended to estimate the level of clustering in a network. Throughout, we consider a population with *N* individuals that interact through some contact process. This population and its interactions are fully described by a undirected random network, denoted *Y*, on *N* nodes. An simple example of a network is shown in Figure 1 with illustrations of some of the terms we use in this article. *Y* can be represented by the symmetric binary matrix [*Y_ij_*] where *Y_ij_* = *Y_ji_* = 1 if an edge is present between nodes *i* and *j*, otherwise *Y_ij_* = 0. We stipulate that there no loops in the network, so *Y_ii_* = 0 for all *i*. The *degree* of the *i*th node, denoted *d_i_* is the number of edges connected to *i*, so *d_i_* = ∑*_j:j>i_* *Y_ij_*.

Clustering is one of the central features of observed social networks [12,13]. Intuitively, clustering is the propensity for triangles or other small cycles to form, so that, for example, a friend of my friend is also likely to be my friend. Where there is a positive clustering effect, the existence of edges (*i, j*) and (*i, k*) increases the propensity for the edge (*j, k*) to exist, while a negative clustering effect implies that (*j, k*) is less likely to exist given the presence of (*i, j*) and (*i, k*). When there is no clustering effect, the presence or absence of (*i, j*) and (*i, k*) has no bearing on that of (*j, k*). Thus clustering is one of the most basic of the true network effects—when it is present, the relationship between two nodes depends not only on properties of the nodes themselves but the presence or absence of other relationships in the network.

The effect of clustering on the dynamics of stochastic epidemics that run over networks remains largely unknown, though it has been studied in a few special cases. The difficulty with studying this effect in isolation is in trying to construct a network model where clustering can change but other properties of the network are held constant. In simulations we study here, we focus on holding the degree sequence of a network constant—that is, each node maintains the same number of contacts—while varying the level of clustering. Intuition suggests that clustering will have some effect on epidemic dynamics since, in a graph with no cycles, if an infection is introduced to a population at node *i* and there is a path leading to *j* then *k*, *k* can only become infected if *j* does first. However, where cycles are present, there may be multiple paths leading from *i* to *k* that do not include *j*, so giving a different probability that *k* becomes infected and a different expected time to infection for *k*.

Previous work on the effect of clustering on epidemic dynamics has produced a variety of results which are largely specific to particular types of networks. Newman [14] and Britton *et al.* [15] show that for a class of networks known as random intersection graphs in which individuals belong to one or more overlapping groups and groups form fully connected cliques, an increase in clustering reduces the epidemic threshold, that is, major outbreaks may occur at lower levels of transmissibility in highly clustered networks. Newman [14], using heuristic methods and simulations, suggests that for sufficiently high levels of transmissibility the expected size of an outbreak is smaller in a highly clustered network than it would be in a similar network with lower clustering. These articles show that graphs with different levels of clustering do, at least in some cases, have different outbreak probabilities and final size distributions for epidemic outbreaks.

Kiss and Green [16] provide a succinct rebuttal to the suggestion that the effects found by [14] and [15] are solely due to clustering. They show that, while the mean degree of the network is preserved in the random intersection graph, the degree distribution varies greatly (in particular, there are many zero-degree nodes) and variance of this distribution increases with clustering. An increase in the variance of the degree distribution has previously been shown to lower the epidemic threshold. They demonstrate that a rewiring of random intersection graphs that preserves the degree sequence but decreases clustering produces networks with similarly lowered epidemic thresholds and even smaller mean outbreak sizes. Our experiments, reported below, are similar in spirit to those of [16] but look at networks with different degree distributions and study in detail how epidemic data from networks with varying levels of clustering might vary.

Ball *et al.* [17] show, using analytical techniques, that clustering induced by household structure in a population (where individuals have many contacts with individuals in the same household and fewer global contacts with those outside of the household) has an effect on probability of an outbreak and the expected size of any outbreak. The probability of an outbreak, in some special cases, is shown to be monotonically decreasing with clustering coefficient and the expected outbreak size also decreases with clustering. There is no suggestion that these results will apply to clustered networks outside of this specific type of network or that they apply when degree distributions are held constant.

Eames [18] also studies networks with two types of contacts: regular contacts (between people who live or work together, for example) and random contacts (sharing a train ride, for example). Using simulations of a stochastic epidemic model and deterministic approximations, it is shown that both outbreak final size and probability of an outbreak are reduced with increased clustering, particularly when regular contacts dominate. As the number of random contacts increases, the effect of clustering reduces to almost zero. Strong effects on the expected outbreak size in networks with no random contacts are observed for values of the clustering coefficient above about 0.4, however, no indication of the magnitude of the variance of these effects is given.

Keeling [19] reports similar results, introducing clustering to a network using a spatial technique—nodes live in a two-dimensional space and two nodes are connected by an edge with a probability inversely proportional to their distance. The clustering comes about by randomly choosing positions in space to which nodes are attracted before connections are made. The results suggest that changes in clustering at lower levels has little effect on the probability of an outbreak, but as the clustering coefficient reaches about 0.45, the chance of an outbreak reduces significantly. As in [14] and [15], while the mean degree of network nodes is held constant here, nothing is said about the degree distribution as clustering varies.

Serrano and Boguñá [20] look specifically at infinite power-law networks and shows that the probability of an outbreak increases as clustering increases but the expected size of an outbreak decreases.

Some more recent papers seek to distinguish the effects of clustering from confounding factors such as assortativity and degree sequence. Miller [21] develops analytic approximations to study the interplay of various effects such as clustering, heterogeneity in host infectiousness and susceptibility and the weighting of contacts on the spread of disease over a network. The impact of clustering on the probability and size of an outbreak is found to be small on “reasonable” networks so long as the average degree of the network is not too low. The rate at which the epidemic spreads, measured by the reproduction number, *R*_0_, is found to reduce with increased clustering in such networks. In networks with low mean degree, *R*_0_ may be reduced to point of affecting the probability and size of an outbreak.

Miller [22] points out that studies of the effects of clustering should take into account assortativity in the network, that is, the correlations in node degree between connected nodes. Assortativity has been shown to affect epidemic dynamics and changing the level of clustering in a network can change the level of assortativity. To distinguish between the effects of assortativity and clustering, a method of producing networks with arbitrary degree distributions and arbitrary levels of clustering with or without correlated degrees is presented and studied using percolation methods. The effect of increasing clustering in these models is to reduce the probability of outbreaks and reduce the expected size of an epidemic. Badham and Stocker [23] use simulated networks and epidemics to study the relationship between assortativity and clustering. Their results suggest that increased clustering diminished the final size of the epidemic, while the effect of clustering on probability of outbreak was not very clear. Like [23], Moslonka-Lefebvre *et al.* [24] use simulations to try to distinguish the effects of clustering and assortativity but look at directed graphs. Here, they find that clustering has little effect on epidemic behaviour.

Melnik *et al.* [25] propose that the theory developed for epidemics on unclustered (tree-like) networks applies with a high degree of accuracy to networks with clustering so long as the network has a small-world property [12]. That is, if the mean length of the shortest path between vertices of the clustered network is sufficiently small, quantities such as the probability of an outbreak on the network can be estimated using known results that require only the degree distribution and degree correlations. The theory is tested using simulations on various empirical networks from a wide range of domains and synthetic networks simulated from theoretical models.

Taken together, these studies show that clustering can have significant effects on crucial properties of epidemics on networks such as the probability, size and speed of an outbreak. These results primarily relate to the final outcome and mean behaviour of epidemics. However, if we can obtain a transmission tree for an outbreak then we have information from the start to the finish of a particular epidemic including times of infection and who infected whom. Since epidemics are stochastic processes, data from a particular epidemic may differ considerably from the predicted mean. Whether or not such data contains information about clustering in the underlying network is the question we seek to address here.

We simulate epidemics over networks with fixed degree distributions and varying levels of clustering and inspect various summary statistics of the resulting epidemic data, comparing the summaries for epidemics run over networks with the same degree distribution but different levels of clustering. The precise details of the simulations are described in Section 2. The results of the simulations, presented in Section 3, show that there is likely little to no signal of clustering in a contact network to be found in a single realisation of an epidemic process over that network.

We conclude that it is unlikely that clustering parameters can be inferred solely from epidemiological data that relates to the transmission tree and suggest that further work in parameter estimation for contact networks would be best focused on other properties of contact networks such as degree distribution or broader notions of population structure.

## 2. Methods

### 2.1. Simulating Networks and Measuring Clustering

We simulate multiple networks from two network models: a Bernoulli model [26] and a power-law model [27]. Under the Bernoulli model (also called the Erdős-Rényi or binomial model), an edge between nodes *i* and *j* is present with some fixed probability 0 ≤ *p* ≤ 1 and absent with probability 1 – *p*, independently of all other edges. Due to their simplicity, Bernoulli networks are well-studied and commonly used in disease modeling but are not generally thought to be accurate models of social systems. A Bernoulli network is trivial to construct by sampling first the total number of edges in a the graph |*Y* | = ∑*_i>j_* *Y_ij_* ∼ Binomial(*N*(*N* – 1)*/*2, *p*), where *N* is the number of nodes in the network, and then sampling |*Y* | edges uniformly at random without replacement. We set *N* = 500 and *p* = 7*/N* = 0.014 in the simulations reported below.

A power-law network is defined as having a power-law degree distribution, that is, for nodes *i* = 1*, …, N*, *P* (*d_i_* = *k*) *∞ k^−α^* for some *α >* 0. Power-law networks are commonly used to model social interactions and various estimates of *α* in the range 1.5–2.5 have been claimed for observed social networks. In the model used here, we set *α* = 1.8. We simulate power-law using a Reed-Molloy type algorithm [28]. That is, the degree of each node, *d_i_*, *i* = 1*, …, N*, is sampled from the appropriate distribution. Node *i* is then assigned *d_i_* “edge stubs” and pairs of stubs are sampled uniformly without replacement to be joined and become edges. When all stubs have been paired, loops are removed and multiple edges between the same nodes are collapsed to single edges. This last step of removing loops and multiple edges causes the resulting graph to be only an approximation of a power law graph but the approximation is good for even moderately large *N*. We set *N* = 600 and consider only the largest connected component of the network in the simulation reported below.

The size of the networks considered here is smaller than some considered in simulation studies though on a par with others (see, for example, [25] who looks a a wide range of network sizes). We choose these network sizes partly for convenience and partly because the current computational methods for statistical fitting of epidemic data to network models would struggle with networks much larger than a few hundred nodes [6] so our interest is in networks around this size.

From each sampled network, *Y*, we generate two further networks, *Y^hi^* and *Y^lo^* that preserve the degrees of all nodes in *Y* but have, respectively, high and low levels of clustering. We achieve this using a Monte Carlo algorithm implemented in the ERGM package [29] in R [30] that randomly rewires the input network while preserving the degree distribution. A similar algorithm is implemented in Bansal *et al.* [31]. For details of the ERGM model and implementation of this algorithm, we refer the reader the package manual [32] and note that the two commands used to simulate our networks are

y_hi = simulate(y ∼ gwesp (0.2, fixed=T), theta0 = 5,...

  constraints = ∼ degreedist, burnin=5e+5)

and

y_lo = simulate(y ∼ gwesp(0.2, fixed=T), theta0 = −5,...

  constraints = ∼ degreedist, burnin=5e+5)

We measure clustering in the resulting networks using the clustering coefficient [12], defined as follows. Let *N_i_* = {*j*|*Y_ij_* = 1} be the neighbourhood of vertex *i* and *d_i_* = |*N_i_*| be the degree of *i*. Let *n_i_* = ∑_*j<k*∈*N*_*i*__ *Y_jk_* be the number of edges between neighbours of *i*. Then, if *d_i_* *>* 1, the local clustering coefficient is *C_i_* = 2*n_i_/d_i_*(*d_i_* – 1), which is the ratio of extant edges between neighbours of *i* to possible edges. For *d_i_* ∈ {0*,* 1}, let *C_i_* = 0. The (global) clustering coefficient is the mean of the local coefficients, $C=\sum_{i=1}^{N}C_{i}/N$. The choice of *C_i_* = 0 for *d_i_* ∈ {0*,* 1} is somewhat arbitrary, though other possible choices, such as *C_i_* = 1 or excluding those statistics from the mean, give similar qualitative results in our experiments.

### 2.2. Simulating Epidemics

Over each simulated network, we simulate a stochastic susceptible-exposed-infectious-removed (SEIR) epidemic. All nodes are initially susceptible to the infection. The outbreak starts when a single node is chosen uniformly at random and exposed to a disease. After a gamma-distributed waiting period with mean *k_E_θ_E_* and variance $k_{E}\theta_{E}^{2}$, the node becomes infectious. The infection may spread across the edges of the network, from infectious nodes to susceptible nodes according to a Poisson process with rate *β*. Infected nodes recover after an infectious period with a gamma distributed waiting time with mean *k_I_θ_I_* and variance $k_{I}\theta_{I}^{2}$. Once a node is recovered, it plays no further part in the spread of the infection. The process stops when there are no longer any exposed or infectious nodes. For each pair, *Y^hi^* and *Y^lo^*, we start the infection from the same node. We condition on the outbreak infecting at least 20 nodes. The parameter values are set at *β* = 0.1, *k_E_* = *k_I_* = 1 and *θ_E_* = *β_I_* = 3 in the simulations reported below.

### 2.3. Summarising Epidemic Data

A transmission tree encodes all information about the epidemic outbreak it describes. As such, it is a very complicated object. To compare sets of transmission trees and decide whether there are some systematic differences between them, we rely on various summary statistics derived from the trees and compare the distribution of the summaries over the ensembles in question. The summaries we use can be divided into two groups, those relating solely to the number of infected through time and those relating to topology of the tree.

The first group of summaries can all be derived from the epidemic curves, that is, the number infected as a function of time. From this, we derive scalar summaries being the total number of individuals infected, the length of the epidemic (measured from the time of the first infection to the last recovery), the maximum of the epidemic curve and the time of that maximum.

**Figure 2.**
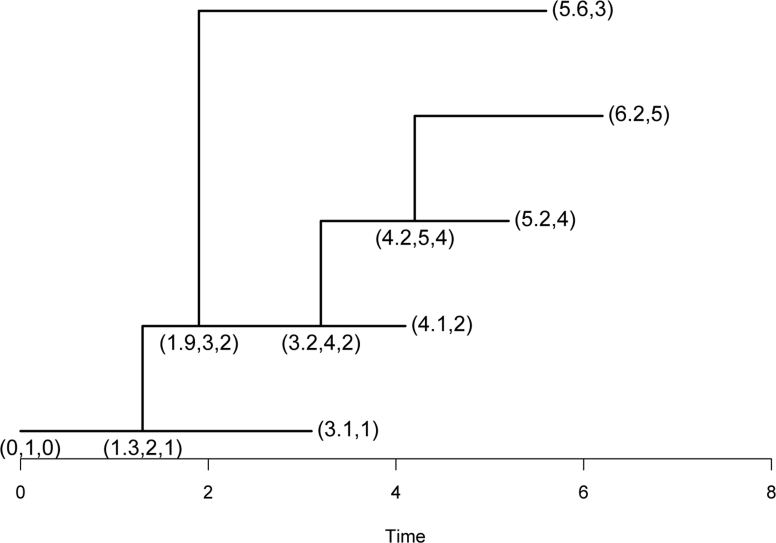
An example of transmission tree showing the labelling scheme. Five individuals are involved in the epidemic and are labelled 1*, …,* 5. The root node is labelled (0*,* 1*,* 0) to signify that, at time *t* = 0, individual 1 was (spontaneously) infected. The internal nodes represent transmission events via a triplet such as (1.3*,* 2*,* 1) showing that, at time *t* = 1.3, individual 2 was infected by individual 1. The leaf nodes represent recovery times, for example (3.1*,* 1) means that, at time *t* = 3.1, individual 1 recovered. Note that this tree has one “cherry”, formed by the leaves labelled (5.2*,* 4) and (6.2*,* 5), out of a possible maximum of two cherries.

We label each individual in the population (equivalently, each node in the contact network) with labels 1, …, *N*. A transmission tree, a distinct graph from the contact network, has a time component and can be defined as follows; an example of a transmission tree and the notation is given in Figure 2. There are three types of nodes in a transmission tree (not to be confused with nodes in the contact network): the root node corresponding to the initial infection, transmission or internal nodes corresponding to transmission events, and leaf or external nodes corresponding to recovery events. Leaf nodes are defined by the time and label pair (*t_i_, u_i_*) where *t* ≥ 0 is the time of the recovery event and *u_i_* is the label of individual that recovered. The internal nodes are associated with the triple (*t_i_, u_i_, v_i_*) being the time of the event, *t_i_*, the label *u_i_* of the exposed individual and *v_i_* that is the transmitter or “parent” of the infection. The root node is like an internal node but the infection parent is given as 0, so is denoted (*t*_0_*, u*_0_*,* 0). The branches of the tree are times between infection, transmission and recovery events for a particular vertex. For example, if the individual labelled *u* is infected at event (*t*_1_*, u, v*_1_), is involved in transmission events (*t_k_, v_k_, u*), *k* = 2*, …, m* – 1, and recovers at (*t_m_, u*) where *t_i_* *< t_j_* for *i < j* and {*v*_1_*, …, u_m_*_−1_} are other individuals in the population, there are *m* – 1 branches of the transmission tree at *u* defined by the intervals (*t_i_, t_i_*_+1_], for *i* = 1*, …, m* – 1.

We summarise the transmission tree using the following statistics: the mean branch length between internal nodes (corresponding to the mean time between secondary infections for each individual); the mean branch length of those branches adjacent to a leaf node (which corresponds to the mean time from the last secondary infection to removal for each individual); the number of secondary infections caused by each infected individual (that is, for each infected individual *v* we count the number of internal nodes that have the form (*t_i_, u_i_, v*), for some *i*); and, the distribution of infective descendants for each individual, *v*, which is defined recursively as the sum of secondary infections caused by *v* and the secondary infections caused by the secondary infections of *v* and so on. An equivalent definition is to say that number of infective descendants of *v* is the number of leaves that have a node of the form (*t, u_i_, v*) as an ancestor. Finally, we consider the number of cherries in the tree [33] which is the number of pairs of leaves that are adjacent to a common internal node. This simple statistic is chosen as it is easy to compute and contains information about the topology or shape of the tree. To compare the number of cherries in outbreaks of different size, we look at the ratio of extant cherries to the maximum possible number of cherries for the given outbreak.

The experimental pipeline can thus be summarised as:

1. Repeat for *i* = 1*, …,* 500:
  1. Sample a graph *Y_i_* according to given degree distribution.
  2. Simulate two further graphs $Y_{i}^{hi}$ and $Y_{i}^{lo}$ with high clustering and low clustering, respectively, using a Monte Carlo sampler that rewires *Y_i_* to alter the clustering level while preserving the degree of each node.
  3. Simulate SEIR epidemics over $Y_{i}^{hi}$ and $Y_{i}^{lo}$, conditioning on a major outbreak occurring in each.
  4. Extract resulting transmission trees from $Y_{i}^{hi}$ and $Y_{i}^{lo}$ and calculate the respective summaries, $S_{i}^{hi}$ and mm $S_{i}^{lo}$.
  5. Compare sets of summaries, *S^hi^* and *S^lo^*.

## 3. Results

We report results here for SEIR epidemics run over Bernoulli and power-law networks. A number of smaller trials that we do not report were run: with different values chosen for the network and epidemic parameters; on networks with the same degree distributions as a random intersection graph; and, using an SIR epidemic rather than an SEIR. The results for those smaller trials were qualitatively similar to the results reported here.

**Figure 3.**
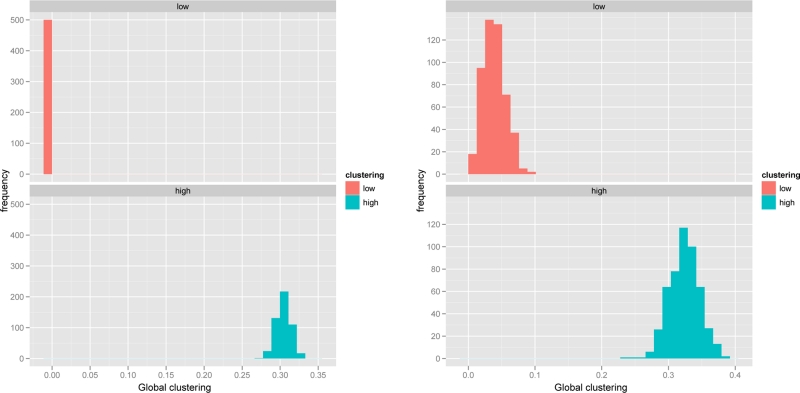
Clustering coefficient for (left) Bernoulli networks and (right) power-law networks.

The distributions of the measured clustering coefficients is shown in Figure 3 and show that the simulated networks with high and low clustering for a given degree distribution are easily distinguished from one another. The Bernoulli networks with low clustering contain no triangles, so the clustering coefficient for each of these networks is zero, while for highly-clustered Bernoulli networks, clustering coefficients are in the range (0.28,0.33). For the power-law networks, the low clustered networks have clustering in the range (0.00,0.09) while the highly clustered networks have clustering in the range (0.24,0.38).

**Figure 4.**
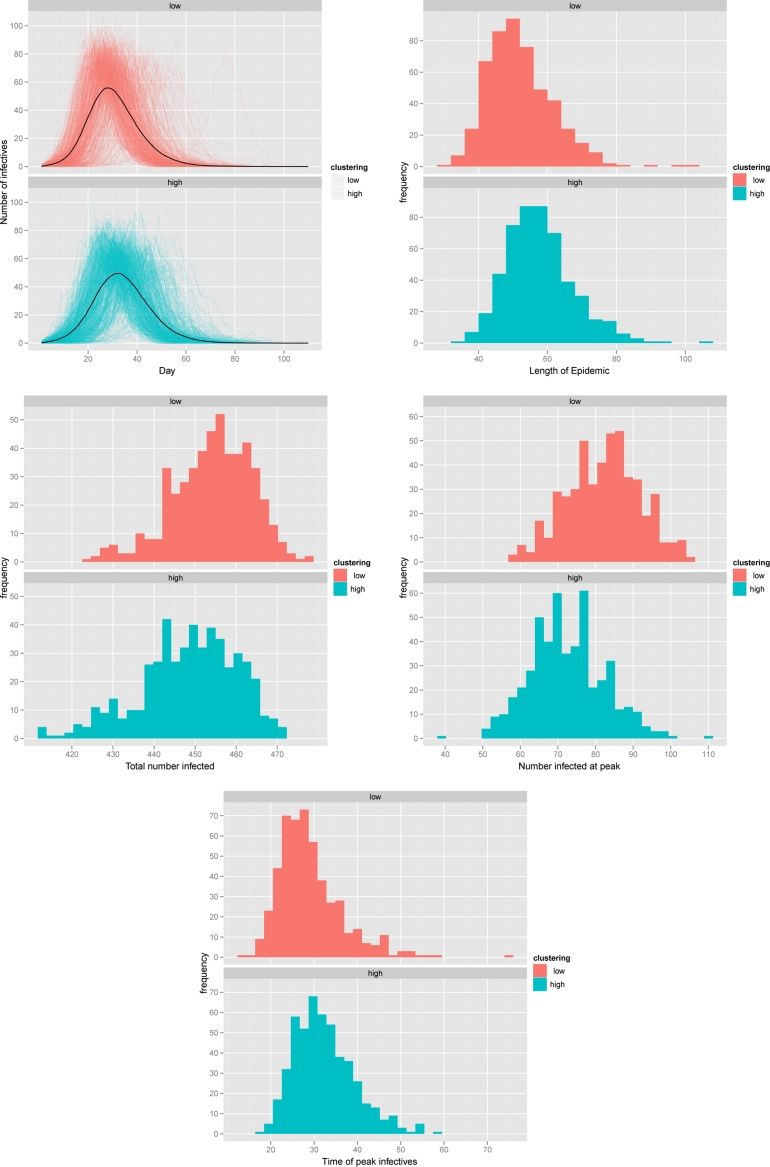
Empirical distributions of summary statistics for epidemics on Bernoulli networks. (top left) Number of infected individuals through time with daily mean shown in black; (top right) Length of epidemic; (centre left) Maximum number infected at peak of outbreak; and, (bottom) Time of outbreak peak.

**Figure 5.**
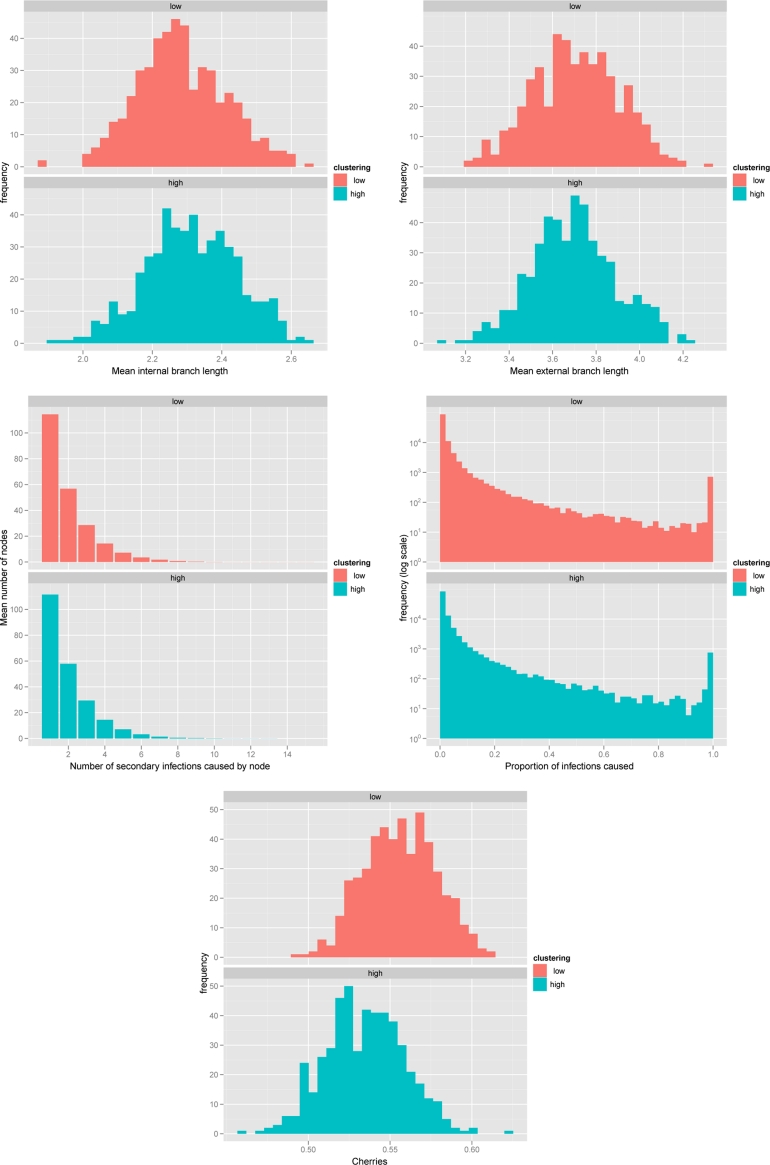
Empirical distributions of summary statistics of transmission trees from epidemics on Bernoulli networks. (top left) Mean internal branch length; (top right) Mean external branch length; (middle left) Number of secondary infections by node; (middle right) Number of total infections by node, vertical axis on log-scale; and, (bottom) Number of cherries in tree as a proportion of possible cherries.

Figures 4 and 5 show comparisons of summary statistics for networks with differing levels of clustering and Bernoulli degree distributions. The summaries show some differences between the outbreaks on the differently clustered networks. In particular, the outbreaks in the highly-clustered networks spread more slowly, on average, leading to marginally longer epidemics with fewer individuals infected at the peak of the outbreak, that occurs slightly later, than we see in outbreaks on the networks with low clustering. These mean effects are in line with the predictions of [22]. The variances of the measured statistics, however, are sufficiently large due to stochastic effects in the model that the ranges of the distributions overlap almost completely in most cases. Statistics derived from the transmission tree appear to add little information, with only the number of cherries differing in the mean.

**Figure 6.**
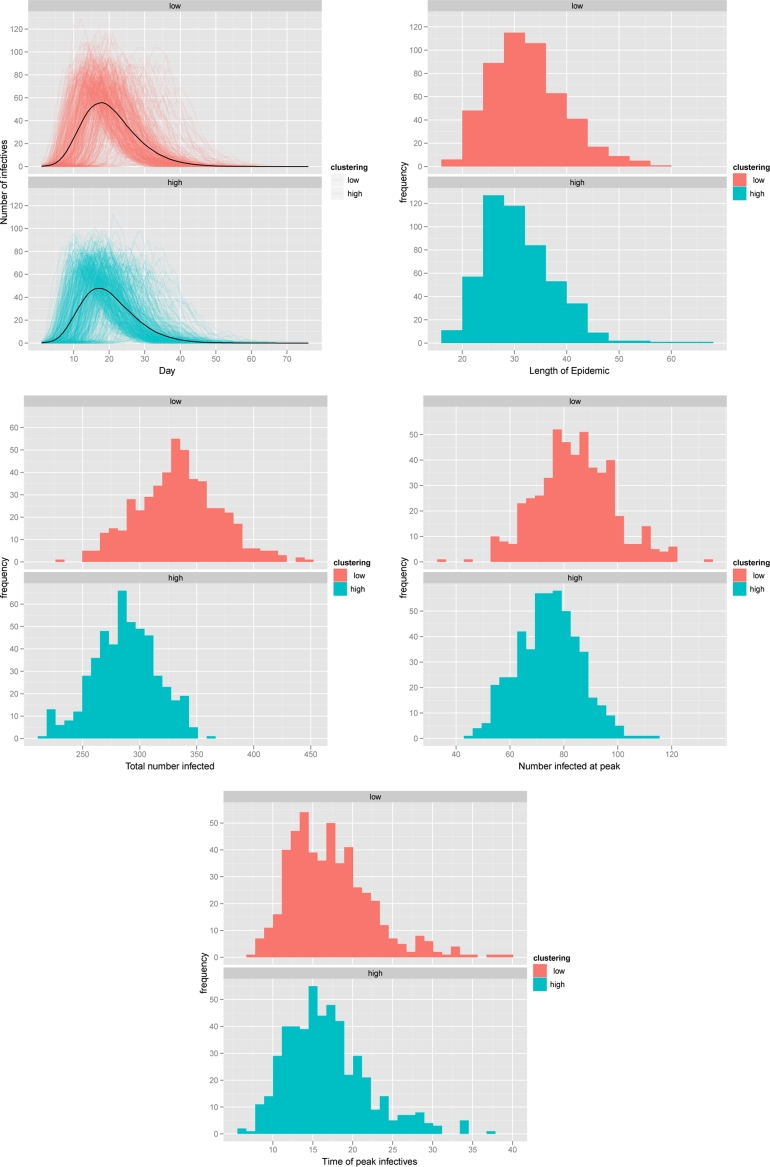
Empirical distributions of summary statistics of epidemics on power-law networks. (top left) Number of infected individuals through time with daily mean shown in black; (top right) Length of epidemic; (centre left) Maximum number infected at peak of outbreak; and, (bottom) Time of outbreak peak

**Figure 7.**
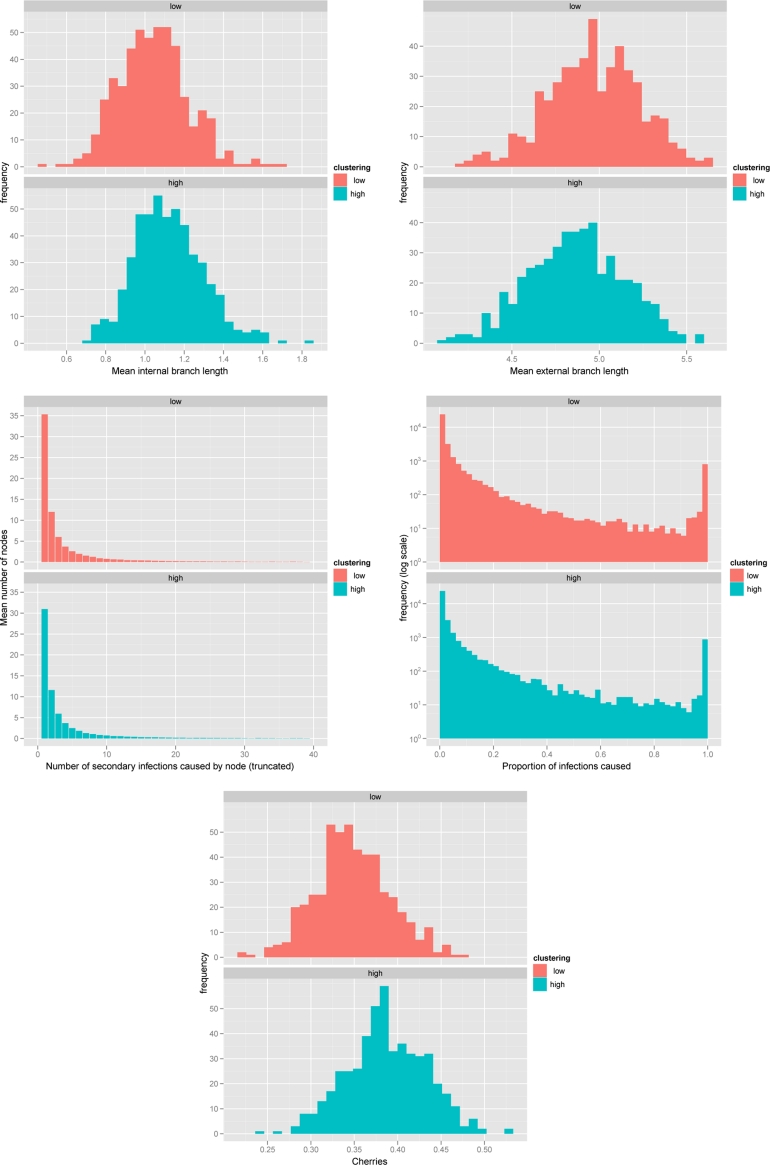
Empirical distributions of summary statistics of transmission trees from epidemics on power-law networks. (top left) Mean internal branch length; (top right) Mean external branch length; (middle left) Number of secondary infections by node; (middle right) Number of total infections by node, vertical axis on log-scale; and, (bottom) Number of cherries in tree as a proportion of possible cherries.

Figures 6 and 7 show the corresponding distributions for networks with power-law degree distributions. Again, differences in the means between the two sets of statistics are apparent with the mean length of epidemic, total number infected and number infected at peak all lower in the epidemics on networks with high-clustering. The largest difference is found in the total number infected, where in the low-clustered networks, the range of the statistic is (231*,* 445) while it is just (211*,* 361) in the high-clustered networks. The primary cause here is due to the change in size of the largest connected component of the network. If we adjust for this by looking instead at the proportion of the giant component infected, the distributions again overlap almost completely with the range for the proportion infected in the low-clustered networks being (0.39*,* 0.74) and (0.42*,* 0.74) for the high-clustered networks.

## 4. Discussion and conclusions

The results presented above suggest that the behaviour of an epidemic on a random network with a given degree sequence is relatively unaffected by the level of clustering in the network. Some effect is seen, but it is small relative to the random variation we see between epidemics on similarly clustered networks. The results also suggest that the complete transmission tree from an epidemic provides little information about clustering that is not present in the epidemic curve. These results do not imply that clustering has little effect, rather they suggest as noted in [16], the apparently strong effect of clustering observed by some is more likely to due to a change in the degree distribution—an effect we have nullified by holding the degree sequence constant. These broader effects are probably best analysed on a grosser level such as the household or subgroup level rather than at the individual level at which clustering is measured.

Our simulation method, in which the degree sequence for each network is held constant while clustering levels are adjusted, places significant restrictions on the space of possible graphs and therefore clustering coefficients. The levels of clustering achieved in the simulations reported here (for example, having a clustering coefficient in the low-clustered Bernoulli case of 0 versus a mean of 0.30 for the high-clustered case) are not so high as those considered in the some of the simulations and theoretical work described in Section 1, and this may partly account for the limited effect on epidemic outcomes that we find here. There is little known about the levels of clustering found in real contact networks [31] (though one recent detailed study [34] find values for clustering in a social contact network in the region 0.15–0.5) and no evidence to suggest that very extreme values of clustering are achieved for a given degree sequence. It is plausible, however, that the degree sequence of a social network of interest could be found—for example, via ego-centric or full-network sampling [34–36]—and therefore reasonable to explore the achievable levels of clustering conditional on the degree sequence. In doing so, we separate the effects on epidemic dynamics of change in the degree sequence of the contact network from those of clustering.

From a statistical point of view, these results indicate that even with full data from a particular epidemic outbreak, such as complete knowledge of the transmission tree, it is unlikely that the level of clustering in the underlying contact network could be accurately inferred independently of the degree distribution. This is primarily due to the large stochastic variation found from one epidemic to the next that masks the relatively modest effects of clustering on an outbreak. With this much stochastic noise, we suggest that it would require data from many outbreaks over the same network (that is, pathogens with a similar mode of transmission spreading in the same population) to infer the clustering level of that network with any accuracy. The results also suggest that attempting to estimate a clustering parameter without either estimating or fixing the degree sequence, as in Goudie [37], may see the estimated clustering parameter acting chiefly a proxy for the degree sequence.

It cannot be ruled out that a statistical method, which takes into account the complete data rather than the summaries we use here, or which takes data from parts of the parameter space that we have not touched on here, could find some signal of clustering from such data. In practise, however, it would be highly unusual to have access to anything approaching complete data. A more realistic data set might include times of onset and recovery from disease symptoms for some individuals in the population and sequences taken from viral genetic material. The noise that characterises such data sets already makes it difficult to accurately reconstruct the transmission tree; this extra uncertainty would likely make any inference of a clustering parameter, in the absence of other information, very difficult.
